# The effect of hypothyroidism on the risk of diabetes and its microvascular complications: a Mendelian randomization study

**DOI:** 10.3389/fendo.2023.1288284

**Published:** 2023-12-05

**Authors:** Ting Fang, Xiaoqing Deng, Jingyi Wang, Fei Han, Xiangyang Liu, Yajin Liu, Bei Sun, Liming Chen

**Affiliations:** ^1^ NHC Key Laboratory of Hormones and Development, Tianjin Medical University Chu Hsien-I Memorial Hospital and Tianjin Institute of Endocrinology, Tianjin Medical University, Tianjin, China; ^2^ Tianjin Key Laboratory of Metabolic Diseases, Tianjin Medical University Chu Hsien-I Memorial Hospital and Tianjin Institute of Endocrinology, Tianjin Medical University, Tianjin, China

**Keywords:** Mendelian randomization, diabetes, microvascular complications, diabetic kidney disease, diabetic retinopathy, hypothyroidism

## Abstract

**Context:**

Several observational studies have found that hypothyroidism is associated with diabetes and its microvascular complications. However, the cause and effect have not been clarified.

**Objective:**

The aim of the study was to examine the causality of such associations by a Mendelian randomization study.

**Methods:**

Two-sample Mendelian randomization analysis was conducted to investigate the associations. Summary statistics for hypothyroidism were from the UK Biobank, and diabetes and its microvascular complications were from the largest available genome-wide association studies. MR–Egger, weighted median, inverse variance weighted, simple mode and weighted mode were used to examine the causal associations, and several sensitivity analyses were used to assess pleiotropy.

**Results:**

Inverse variance weighted estimates suggested that hypothyroidism was associated with type 1 diabetes and type 1 diabetes with renal complications (β= 9.059926, se= 1.762903, P = 2.76E-07 and β= 10.18375, se= 2.021879, P = 4.73E-07, respectively) but not type 2 diabetes and type 2 diabetes with renal complications. In addition, hypothyroidism was positively associated with severe nonproliferative diabetic retinopathy and proliferative diabetic retinopathy (β= 8.427943, se= 2.142493, P = 8.36E-05 and β= 3.100939, se= 0.74956, P=3.52E-05, respectively).

**Conclusions:**

The study identified the causal roles of hypothyroidism in diabetes and its microvascular complications. Hypothyroidism can lead to type 1 diabetes, type 1 diabetes with renal complications, severe nonproliferative diabetic retinopathy and proliferative diabetic retinopathy.

## Introduction

Diabetes is a growing public health problem with high health care costs and morbidity. According to data reported by the International Diabetes Federation (IDF), China has the largest number of diabetic patients, with an estimated 141 million adults living with the disease in 2021 and a projected 174.4 million by 2045 (1). Diabetic microvascular complications are the most common complications of diabetes, mainly diabetic kidney disease and diabetic retinopathy. Diabetic kidney disease (DKD) is the most common cause of chronic kidney disease (CKD) and end-stage renal disease (ESRD) worldwide and leads to enormous labor and societal costs (2, 3). Proteinuria and reduced renal function are significant clinicopathological features of DKD in diabetic patients (4). Typical pathological features include impaired endothelial cell function, podocyte disease, glomerular mesangial expansion, basement membrane thickening, tubular sclerosis and tubular interstitial fibrosis (5). Fibrosis, oxidative stress and apoptosis are the major contributing factors in the pathophysiology of renal injury in DKD (6). Diabetic retinopathy is the leading cause of blindness in diabetic patients and is further divided into nonproliferative retinopathy (NPDR) and proliferative retinopathy (PDR). Diabetic retinopathy is caused by metabolic abnormalities (7). Typical pathophysiology includes a thickened retinal capillary basement membrane, increased vascular permeability, tissue ischemia release of various vasoactive substances and neovascularization (8). NPDR is typically characterized by microaneurysm formation and small dilatation of retinal blood vessels, while PDR is characterized by neovascularization.

Hypothyroidism is a systemic hypometabolic syndrome caused by various causes of hypothyroid hormoneemia or thyroid hormone resistance (9). The pathology is characterized by the accumulation of mucopolysaccharides in the tissues and skin, which manifests as mucinous edema. The diagnosis of hypothyroidism depends on elevated serum TSH levels. The main causes of hypothyroidism are autoimmune impairment, thyroid destruction, iodine overdose and the use of antithyroid medications. Hypothyroidism causes pericardial effusion by increasing epicardial vascular permeability and decreasing albumin lymphatic drainage, leading to effusion in the pericardial cavity (10). Treatment of hypothyroidism is aimed at restoring normal thyroid function.

Thyroid dysfunction (TD) and diabetes mellitus (DM) are the two most common chronic endocrine disorders with different prevalences in different populations (11). Studies have reported an increased prevalence of diabetes and its complications in patients with hypothyroidism and vice versa (12). Whether there is a causal relationship between hypothyroidism and diabetes mellitus and its complications deserves further investigation. Mendelian randomization is a recently emerged method for exploring causal relationships. It avoids artificial random grouping and uses genetic variation significantly associated with exposure as an instrumental variable (IV) to infer the causal relationship between exposure and outcome. The genotype corresponding to an individual’s IV locus reflects the level of exposure, and the parental alleles are randomly assigned to the offspring during the deceleration division; thus, the MR method is also known as a “natural randomized controlled trial” and avoids confounding bias and reverse causation, which are common in observational studies. MR has been widely used to explore causal relationships between diseases. In this study, a two-sample MR analysis was performed using genome-wide association study (GWAS) summary statistics from the UK Biobank and FinnGen consortium to assess the causal relationship between hypothyroidism and diabetes and its microvascular complications.

## Materials and methods

### Data sources

Genetic variants for hypothyroidism were obtained from the UK Biobank (ID: ukb-a-77). Type 1 diabetes(T1DM) (ID: finn-b-E4_DM1_STRICT), type 2 diabetes(T2DM) (ID: finn-b-E4_DM2_STRICT), type 1 diabetes with renal complications(T1DM- REN) (ID: finn-b-E4_DM1REN), type 2 diabetes with renal complications(T2DM- REN) (ID: finn-b-E4_DM2REN), severe nonproliferative diabetic retinopathy(NPDR) (ID: finn-b-DM_BCKGRND_RETINA_NONPROLIF) and proliferative diabetic retinopathy(PDR) (ID: finn-b-DM_RETINA_PROLIF) were from the FinnGen consortium. After setting to P < 5 × 10^−8^ and clump=TRUE, r^2^ = 0.001, kb=10000 to remove SNPs that were in linkage disequilibrium, the remaining SNPs were used for MR analysis.

### Statistical analysis

In this study, multiple methods of MR analysis, including MR–Egger regression, weighted median, inverse variance weighted (IVW), simple mode and weighted model, were used to examine whether there was a causal association between hypothyroidism and diabetes and its microvascular complications.

After we calculated the MR results, sensitivity analysis was carried out, which was mainly tested from the following three aspects (1): Heterogeneity test: This mainly tests the differences between SNPs. If SNPs differ greatly from each other, they may have high heterogeneity. The random effects model was used to estimate the MR effect size (2). Pleiotropy test: Pleiotropy mainly tests whether multiple SNPs have horizontal pleiotropy. It is often expressed by the intercept term of the MR Egger method. If the intercept term is very different from 0, it indicates the existence of horizontal pleiotropy (3). Leave-one-out sensitivity test: This test is mainly used to calculate the MR results of the remaining SNPs after the removal of SNPs one by one. If the MR results estimated by other SNPs after the removal of a certain SNP are very different from the total results, it indicates that the MR results are sensitive to the SNP.

The above data were analyzed using R (version 4.0.2) and the TwoSampleMR package.

## Results

### GWAS of hypothyroidism in UK Biobank participants

There are 10,894,596 SNPs associated with hypothyroidism in the UK Biobank. When set to P < 5 × 10^−8^ and clump=TRUE, r^2^ = 0.001, kb=10000 to remove SNPs that were in linkage disequilibrium, 83 SNPs were used for MR analysis (**Table 1**).

**Table 1:** The 83 SNPs associated with hypothyroidism from a GWAS involving UK Biobank participants.

SNP	chr	pos	effect_ allele	other_ allele	eaf	beta	se	pval
rs17020110	1	108354156	C	T	0.267645	0.00413049	0.00058753	2.07E-12
rs6679677	1	114303808	A	C	0.102573	0.0201022	0.000852993	1.07E-122
rs12117927	1	236629134	A	C	0.493528	0.0032687	0.000532232	8.18E-10
rs4081335	1	1140504	C	T	0.0532964	-0.0063816	0.00115775	3.55E-08
rs6426808	1	19835415	A	G	0.509924	0.00327227	0.000520025	3.13E-10
rs926103	1	156784982	C	T	0.652112	-0.00298868	0.000547963	4.92E-08
rs11675342	2	1407628	T	C	0.422209	0.00461109	0.000525295	1.67E-18
rs7582694	2	191970120	G	C	0.773961	-0.00697507	0.000620534	2.61E-29
rs11571297	2	204745003	C	T	0.4907	-0.00859242	0.000521363	5.34E-61
rs2111485	2	163110536	G	A	0.609397	0.00380166	0.000530651	7.84E-13
rs13399762	2	55880308	G	A	0.0541387	0.00672799	0.00114529	4.25E-09
rs1534430	2	12644736	T	C	0.389593	-0.00402779	0.000532667	3.99E-14
rs113229608	3	121743159	A	C	0.0640675	0.00647516	0.00106425	1.17E-09
rs11706511	3	39344438	G	A	0.126089	0.00465932	0.000781286	2.47E-09
rs9815073	3	188115682	A	C	0.347569	-0.00705726	0.000567968	1.94E-35
rs7649344	3	37006396	C	T	0.458522	-0.00296836	0.000521217	1.23E-08
rs13090803	3	105934953	T	G	0.212032	0.0050706	0.00063893	2.09E-15
rs4276275	4	10723685	T	C	0.472519	0.00340427	0.000519895	5.84E-11
rs6833591	4	123546282	G	A	0.347415	-0.00317388	0.000545888	6.10E-09
rs3775291	4	187004074	T	C	0.297729	-0.00403311	0.000566167	1.05E-12
rs4444866	4	40307533	T	C	0.278846	-0.00322868	0.000581693	2.85E-08
rs7441808	4	26090375	G	A	0.302384	0.00354074	0.000564386	3.53E-10
rs13145888	4	149634572	C	T	0.212492	-0.00601133	0.000633817	2.45E-21
rs10036386	5	76543603	T	C	0.381915	0.0032141	0.00053396	1.75E-09
rs244672	5	133419283	T	C	0.877485	-0.00462791	0.000790748	4.84E-09
rs28157	5	102595837	T	G	0.316822	-0.00349175	0.000558853	4.16E-10
rs2736191	6	31560910	G	C	0.0250312	-0.00937316	0.00165851	1.59E-08
rs9497965	6	148521292	T	C	0.408708	0.00364705	0.000528511	5.19E-12
rs4263621	6	435471	A	G	0.537567	0.00295553	0.000521765	1.48E-08
rs9272426	6	32605189	G	A	0.460274	0.0123738	0.000529183	7.96E-121
rs76518703	6	31329466	G	A	0.0546591	-0.0100896	0.00122104	1.42E-16
rs28418426	6	32619654	C	T	0.5296	0.00917276	0.000570025	3.06E-58
rs933243	6	167403873	A	C	0.33412	-0.00557022	0.000549604	3.90E-24
rs7768019	6	31202575	G	C	0.246831	-0.00812346	0.000601065	1.31E-41
rs654537	6	90990050	A	G	0.611191	0.00599281	0.000532298	2.13E-29
rs2523483	6	31353792	G	T	0.0874958	-0.00664685	0.00091758	4.37E-13
rs9277569	6	33058402	T	C	0.106947	0.00840785	0.000839758	1.36E-23
rs761357	6	135902599	T	A	0.376767	0.00331651	0.000537459	6.81E-10
rs221786	7	100266081	C	T	0.886238	0.00494967	0.000819327	1.53E-09
rs60600003	7	37382465	G	T	0.100946	0.00500327	0.000864647	7.19E-09
rs6992869	8	61395832	C	T	0.372952	0.00299144	0.000537158	2.56E-08
rs2921053	8	8319963	C	G	0.453459	-0.00467491	0.000521912	3.34E-19
rs1032129	8	119951900	C	A	0.35438	-0.00302426	0.000543772	2.67E-08
rs16903097	8	129556356	G	T	0.130854	-0.00452308	0.00076851	3.97E-09
rs853303	8	133932818	G	A	0.621756	-0.00374837	0.000534335	2.30E-12
rs10956412	8	129162497	C	A	0.163144	-0.00508727	0.000706129	5.84E-13
rs11783023	8	141639262	T	C	0.720606	-0.00329795	0.000576457	1.06E-08
rs911760	9	5438435	A	C	0.207503	0.00418051	0.000672507	5.10E-10
rs2123340	9	21589041	A	G	0.651292	-0.00373671	0.000546057	7.76E-12
rs925489	9	100546600	T	C	0.668458	0.00985707	0.000550975	1.52E-71
rs11258303	10	6405534	A	C	0.746995	0.00371265	0.000598646	5.59E-10
rs71508903	10	63779871	T	C	0.193712	0.00625717	0.000661691	3.21E-21
rs3850765	10	124139910	C	T	0.585106	0.00313025	0.000526496	2.76E-09
rs7905731	10	64018472	C	T	0.594309	-0.00314417	0.000528315	2.66E-09
rs683763	10	89807680	T	G	0.321202	0.00309064	0.000555044	2.57E-08
rs7090530	10	6110875	A	C	0.603596	0.00418704	0.000530431	2.94E-15
rs6584277	10	101278055	G	A	0.525655	-0.0031819	0.000519066	8.79E-10
rs736374	11	35266944	A	G	0.363007	0.00412144	0.000540637	2.48E-14
rs4409785	11	95311422	C	T	0.171755	0.00647971	0.00068747	4.31E-21
rs3184504	12	111884608	C	T	0.517816	-0.0099309	0.000518629	1.10E-81
rs11052877	12	9905690	G	A	0.371466	-0.00576124	0.000537973	9.31E-27
rs12582330	12	103892941	T	G	0.728871	-0.00416475	0.00058512	1.10E-12
rs705702	12	56390636	G	A	0.33759	0.00367966	0.00054845	1.96E-11
rs66749983	13	43063831	T	A	0.310435	0.00360186	0.000561314	1.39E-10
rs111618453	13	24775726	A	G	0.272172	0.00577379	0.000583397	4.33E-23
rs76428106	13	28604007	C	T	0.0133586	0.027135	0.00238035	4.26E-30
rs1045853	14	106208086	C	A	0.656897	-0.00337461	0.000553686	1.10E-09
rs8008961	14	68752643	T	C	0.27975	-0.00336955	0.000577942	5.54E-09
rs8043085	15	38828140	T	G	0.233733	0.00425081	0.000614119	4.47E-12
rs142997491	16	50729820	G	A	0.0121946	0.0150304	0.00238237	2.81E-10
rs13333582	16	67549547	C	T	0.0427567	0.00722053	0.00128242	1.80E-08
rs8054578	16	79316815	G	A	0.776427	-0.00358256	0.000623096	8.95E-09
rs1088898	17	8876505	T	G	0.768046	0.00338586	0.00061771	4.22E-08
rs61759532	17	7240391	T	C	0.246518	0.00419164	0.000618829	1.26E-11
rs62076510	17	45518583	G	T	0.163945	0.00520033	0.000714773	3.46E-13
rs1790604	18	67518031	G	A	0.536379	-0.00322907	0.000520432	5.49E-10
rs1549142	19	18383794	T	C	0.228817	0.0040914	0.000619147	3.90E-11
rs10424978	19	4837557	A	C	0.59984	-0.0041633	0.00053505	7.21E-15
rs12980063	19	50196992	G	A	0.391004	-0.00339664	0.000532123	1.74E-10
rs2745803	20	17859706	G	A	0.207698	-0.00368215	0.000641164	9.31E-09
rs2823272	21	16798586	A	T	0.31568	-0.00355403	0.000558859	2.03E-10
rs229540	22	37591290	G	T	0.423587	0.00503721	0.000525234	8.83E-22
rs2412974	22	30539821	T	C	0.358692	-0.00311955	0.000540792	8.01E-09

SNP, single-nucleotide polymorphism; chr, chromosome; pos, position; eaf, effect allele frequency in the hypothyroidism genome-wide association study population; rs, reference single-nucleotide polymorphism.

### MR analyses

**Figure 1:**
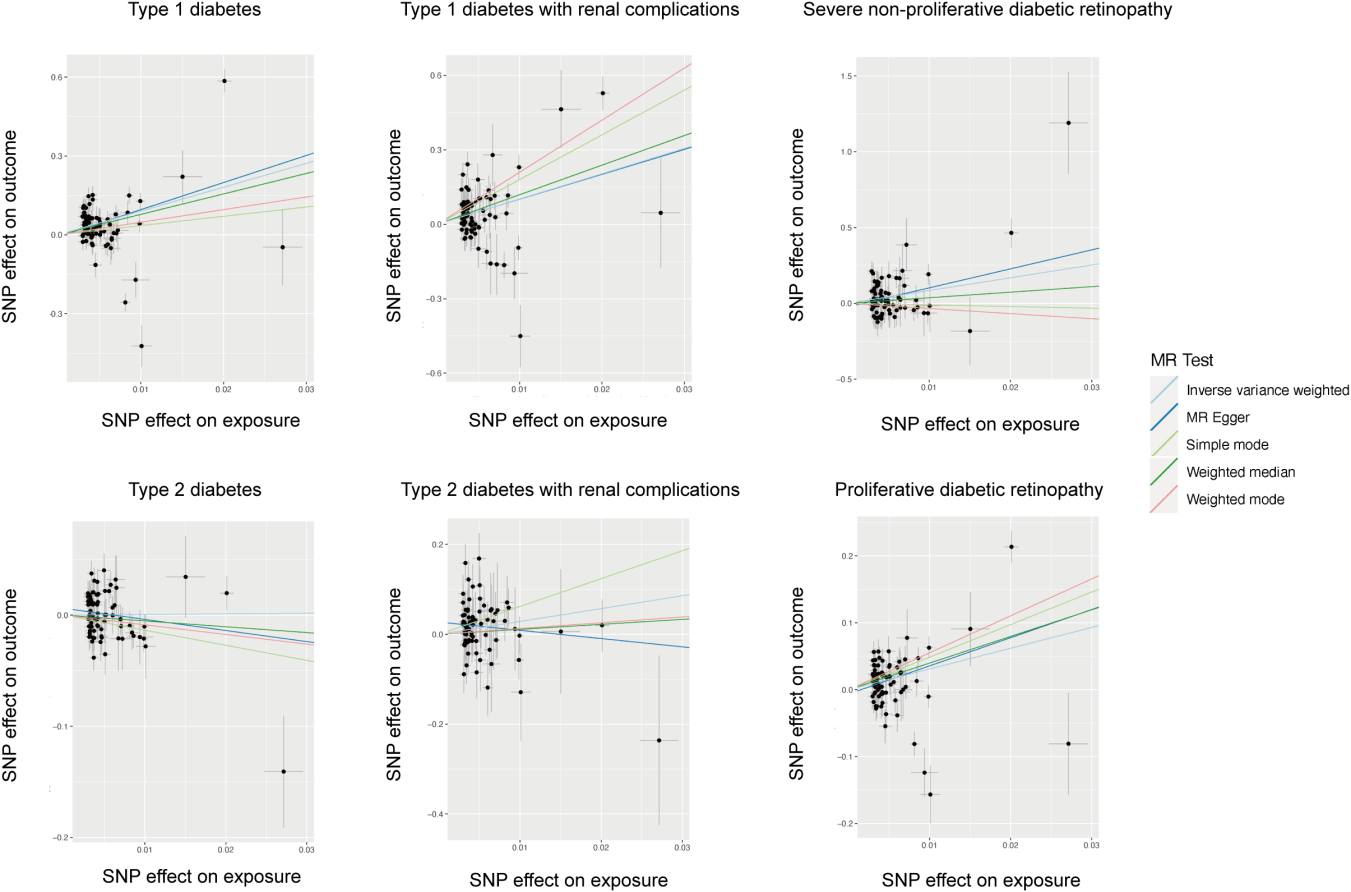
Scatter plot of the causal relationship between hypothyroidism and diabetes mellitus and its microvascular complications.

**Figure 2:**
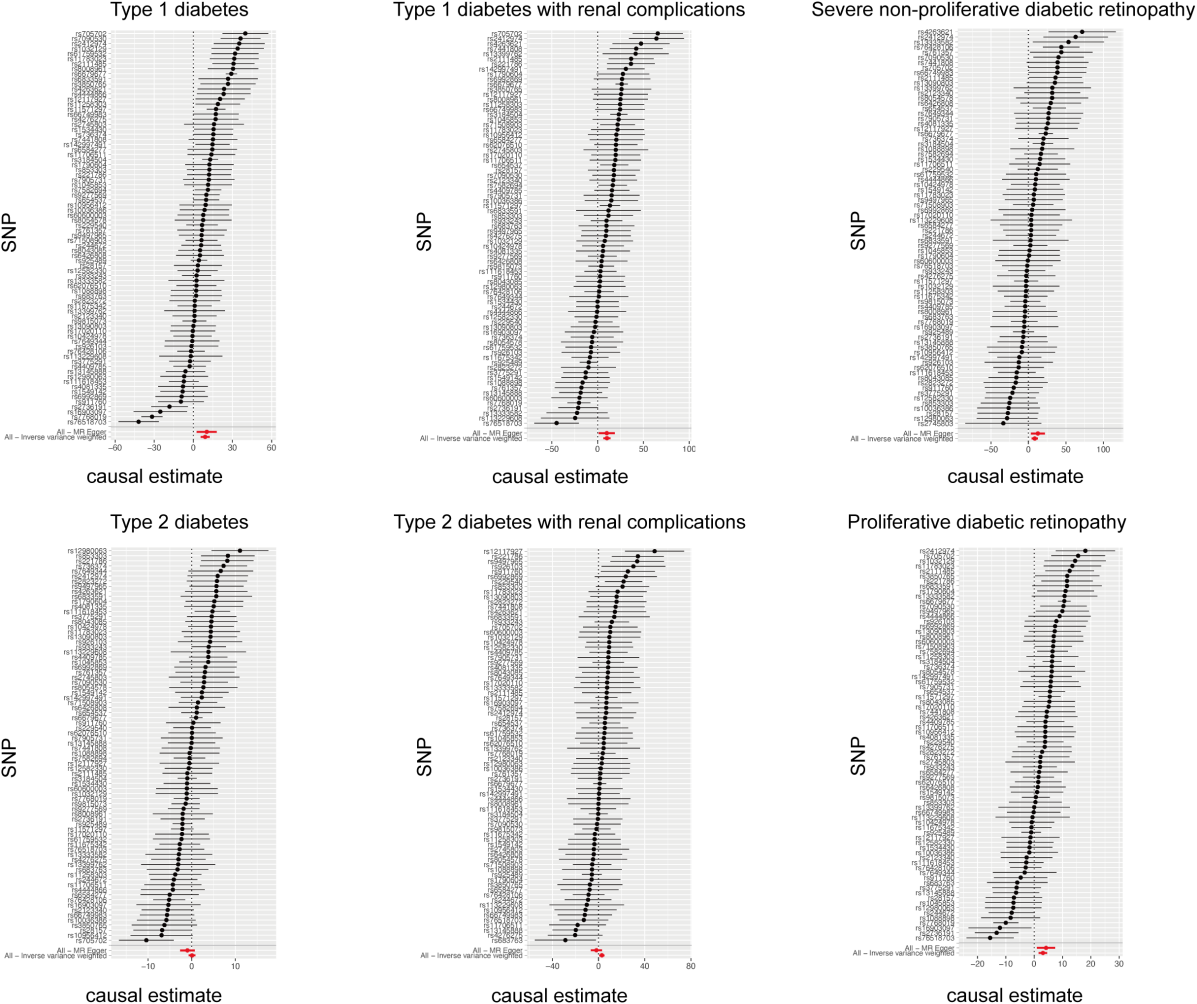
Forest plot of the causal relationship between hypothyroidism and diabetes mellitus and its microvascular complications.

The cause-effect of hypothyroidism on T1DM, T2DM, T1DM-REN, T2DM-REN, NPDR and PDR were estimated by MR analyses (**Table 2**). Scatter plots (**Figure 1**) and forest plots (**Figure 2**) were used to visualize the results of the analysis.

**Table 2:** MR estimates for the association between hypothyroidism and diabetes mellitus and its microvascular complications.

Outcome	Method	NSNP	OR (95%CI)	b	se	pval
T1DM	MR Egger	78	30013.90742(14.9344441, 60319261.46)	10.309416	3.880483	9.61E-03
	Weighted median	78	2402.41909(127.86504734, 45138.35)	7.784231	1.496559	1.98E-07
	Inverse variance weighted	78	8603.51349(271.6828741, 272451.64)	9.059926	1.762903	2.76E-07
	Simple mode	78	34.02116(0.04972605, 23276.31)	3.526983	3.330719	2.93E-01
	Weighted mode	78	121.29389(0.36900206, 39870.26)	4.798216	2.956719	1.09E-01
T2DM	MR Egger	78	0.3684931(0.0702106, 1.933998)	-0.99833329	0.845879	0.2415889
	Weighted median	78	0.5927931(0.22294876, 1.576163)	-0.52290981	0.4989304	0.2946102
	Inverse variance weighted	78	1.0582788(0.49249645, 2.274035)	0.05664379	0.3902611	0.8845977
	Simple mode	78	0.2575864(0.02209098, 3.003523)	-1.35640005	1.2531561	0.2824586
	Weighted mode	78	0.4189852(0.11480456, 1.529108)	-0.86991977	0.6605124	0.1917321
T1DM-REN	MR Egger	78	22515.62(3.551236, 142754000)	10.02196	4.466668	2.78E-02
	Weighted median	78	149984.2(1335.363, 16845810)	11.91829	2.40884	7.51E-07
	Inverse variance weighted	78	26469.47(503.138, 1392526)	10.18375	2.021879	4.73E-07
	Simple mode	78	67462950(245.2726, 18555890000000)	18.02709	6.390163	6.09E-03
	Weighted mode	78	1296053000(585833, 2867291000000)	20.98259	3.929489	9.12E-07
T2DM-REN	MR Egger	78	0.1584635(0.001325609, 18.94275)	-1.842231	2.440639	0.45269264
	Weighted median	78	3.0239249(0.078552654, 116.40755)	1.106556	1.862521	0.55243384
	Inverse variance weighted	78	17.4133103(1.843931204, 164.44397)	2.857235	1.145579	0.01262627
	Simple mode	78	490.4263637(0.609148408, 394843.05461)	6.195275	3.413759	0.07344971
	Weighted mode	78	3.6132542(0.062437441, 209.09899)	1.284609	2.07051	0.53680597
NPDR	MR Egger	78	283686.2(28.3624, 2837484000)	12.555624	4.699265	9.23E-03
	Weighted median	78	39.26462(0.111327, 13848.49)	3.670324	2.992657	2.20E-01
	Inverse variance weighted	78	4573.083(68.62494, 304744.8)	8.427943	2.142493	8.36E-05
	Simple mode	78	0.3418083(0.00000009461703, 1234798)	-1.073505	7.704042	8.90E-01
	Weighted mode	78	0.03364403(0.0000000001078782, 10492580)	-3.39192	9.978622	7.35E-01
PDR	MR Egger	78	67.23067(2.701999, 1672.82187)	4.20813	1.6398662	1.23E-02
	Weighted median	78	53.61646(11.756422, 244.52383)	3.981856	0.7742125	2.70E-07
	Inverse variance weighted	78	22.21881(5.113077, 96.55151)	3.100939	0.74956	3.52E-05
	Simple mode	78	129.89276(1.605567, 10508.5176)	4.866709	2.241445	3.30E-02
	Weighted mode	78	249.60173(1.900938, 32773.83925)	5.519867	2.4885303	2.95E-02

NSNP, number of single nucleotide polymorphism; OR, odds ratio; CI, confidence interval.

### Hypothyroidism and type 1 diabetes

In the IVW MR analysis, the beta value (β) of T1DM for hypothyroidism was 9.059926 (SE= 1.762903; P = 2.76E-07). The heterogeneity test showed that Q pval was much less than 0.05, which indicated strong heterogeneity among these SNPs. Therefore, inverse variance weighted (multiplicative random effects) was used to estimate the MR effect size. According to the results of the random effects model, pval < 0.05 indicates that there is a causal relationship between hypothyroidism and T1DM, and b > 0 indicates that the risk of developing type 1 diabetes increases with increasing hypothyroidism. There was no significant difference between MR–Egger’s Egger intercept and 0 (pval > 0.05), so we can believe that there is no horizontal pleiotropy. All lines are on the right of 0 (**Figure S1**); regardless of which SNP is removed, it will not have a fundamental impact on the result. The leave-one-out result indicates that the MR result is actually robust.

### Hypothyroidism and type 2 diabetes

The beta value (β) of T2DM for hypothyroidism was 0.05664379 (SE= 0.3902611; P = 0.8845977) in the IVW MR analysis. The results of the random effects model showed that pval > 0.05, which indicates that there is no causal relationship between hypothyroidism and T2DM. The leave-one-out result indicates that the MR result is actually robust (**Figure S1**).

### Hypothyroidism and type 1 diabetes with renal complications

The MR analysis of the causal relationship between hypothyroidism and T1DM-REN showed that β was 10.18375 (SE= 2.021879; P = 4.73E-07). The random effects model was used because the Q pval was much less than 0.05 in the heterogeneity test. The result (b= 10.18375, pval = 4.73443e-07) further confirmed the causal relationship between hypothyroidism and T1DM-REN in the random effects model. The risk of developing type 1 diabetes with renal complications increases with increasing hypothyroidism. The pleiotropy test (pval = 0.9676508) proved that there is no horizontal pleiotropy. The MR result is actually robust through the leave-one-out test.

### Hypothyroidism and type 2 diabetes with renal complications

The assessment of causality between hypothyroidism and T2DM-REN was somewhat similar to that of T1DM (β, -1.842231; SE, 2.440639; P = 0.45269264) in the MR Egger analysis. There was no heterogeneity among these SNPs for Q pval = 0.1757848. The MR result that there is no causal relationship between hypothyroidism and T2DM-REN is actually robust through the leave-one-out test.

### Hypothyroidism and severe nonproliferative diabetic retinopathy

The MR analysis of the causal relationship between hypothyroidism and severe nonproliferative diabetic retinopathy (NPDR) showed that β was 8.427943 (SE= 2.142493; P = 8.36E-05). The random effects model was used for the Q pval = 0.001614884 in the heterogeneity test. The result (β= 8.427943, pval = 8.364543e-05) further confirmed the causal relationship between hypothyroidism and NPDR in the random effects model. The pleiotropy test (pval = 0.3267986) proved that there is no horizontal pleiotropy. The MR result is actually robust through the leave-one-out test.

### Hypothyroidism and proliferative diabetic retinopathy

The assessment of causality between hypothyroidism and PDR was somewhat similar to that of NPDR (β, 3.100939; SE= 0.74956; P = 3.52E-05) in the IVW MR analysis. The pleiotropy test (pval = 0.4498041) proved that there is no horizontal pleiotropy. The result (b= 3.100939, pval = 3.518571e-05) further confirmed the causal relationship between hypothyroidism and NPDR in the random effects model. The leave-one-out result indicates that the MR result is actually robust (**Figure S1**).

### Sensitivity analyses

We mainly test from the following three aspects: 1) heterogeneity test, 2) pleiotropy test, and 3) leave-one-out sensitivity test. The heterogeneity test of these six MR results showed that only SNPs of T2DM-REN had no heterogeneity (**Table S1**). Therefore, inverse variance weighted (multiplicative random effects) was used to estimate the MR effect size. The pleiotropy test showed that only SNPs of T2DM-REN have horizontal pleiotropy (**Table S2**). The leave-one-out sensitivity test showed that there was no significant difference between the estimated MR results and the total results of other IVs after removing each IV (**Figure S1**).

## Discussion

Various observational studies have found some interaction between hypothyroidism and diabetes (13, 14). The prevalence of diabetes and its microvascular complications is higher in patients with hypothyroidism than in the general population (15). However, the causal relationship has not been reported. In this study, the causal relationship was analyzed by a Mendelian randomization study, including hypothyroidism and type 1 diabetes, type 2 diabetes, type 1 diabetes with renal complications, type 2 diabetes with renal complications, severe nonproliferative diabetic retinopathy and proliferative diabetic retinopathy.

T1DM is a group of diseases that develop as a result of absolute insulin deficiency due to autoimmune beta cell destruction. In addition, immune function plays an important role in the pathogenesis of hypothyroidism. Joint susceptibility genes identified for T1DM and autoimmune thyroid diseases, including hypo- or hyperthyroidism, are HLA class II genes, PTPN22, CTLA-4 and FOXP3. Candidate genes with joint risk for AITD and T1D are IL-1RA, IL-4, MICA, TNF-α, TG, IL2RA/CD25, VNTR (insulin), ERBB3, CLEC16A and CD40 (16). In our analyses of the causal effect of hypothyroidism on T1DM, there are 78 SNPs involving 56 genes listed below: PDE8B, TNFRSF11B, MIR1208, SPATA13, ILDR1, TPO, AGO2, EDARADD, C12orf42, CPT1C, LOC107986195, PNPT1, NOD2, MIR3681HG, IQCN, LINC00824, VAV3, LOC105373724, C1QTNF6, HORMAD2, VDAC1, NCR3, LOC105372548, MACIR, LOC101927745, LOC107986913, SH2B3, TLR3, PLEKHA1, TNFRSF18, CLNK, LINC02265, ELMO1, ACAP1, EFCAB13, LOC105376819, BACH2, PHTF1, IL21-AS1, LOC105378414, RAB5B, ARID5B, LINC02357, STAT4, AHI1-DT, FLT3, RTKN2, RAD51B, RASGRP1, MAF, TG, PLGRKT, PTCSC2, SH2D2A, SASH1 and LPP. Our analysis revealed additional the above 56 susceptibility genes with one gene previously descibed (TG). This suggests that both are extremely similar in immunogenetics. Our findings reveal a causal relationship between hypothyroidism and T1DM. The limitation of the study is that subclinical hypothyroidism is more frequent than overt disease and is often asymptomatic and thus, undiagnosed and the genetic variants associated with it remain poorly characterized. Our analysis only covered cases with diagnosed hypothyroidism. GWAS data including subclinical hypothyroidism should be addressed in future studies.

On the other hand, T2DM develops as a result of a relative lack of insulin due to insulin resistance (17). Some studies have reported an increased risk of T2DM in patients with hypothyroidism (18, 19). Serum TSH levels were positively correlated with the degree of hyperglycemia and insulin resistance (20–22). In addition, the Rotterdam Study, a large prospective cohort study, also showed that higher TSH levels and lower FT4 levels were associated with the risk of T2DM. In contrast, high and high-normal thyroid function may prevent the development of T2DM (23). Leptin levels were found to correlate with TSH levels and were elevated in patients with hypothyroidism (24). TSH stimulates leptin secretion in adipose tissue (25, 26), increases hepatic glucose output by increasing glucose transporter protein (GLUT) 2 expression in the liver, and stimulates endogenous glucose production by increasing gluconeogenesis and glycogenolysis to reduce hepatic sensitivity to insulin (27). More importantly, TSH decreases beta cell insulin secretion and synthesis, thereby increasing blood glucose. Moreover, increased inflammatory markers in serum, which characterized with T2DM (28), have been reported as a feature of hypothyroidism (29). This suggests that the inflammatory response is enhanced in both hypothyroidism and diabetic patients, and current research suggests that the two interact through inflammation. This is consistent with our result that there is no causal relationship between hypothyroidism and T2DM.

In addition to diabetes itself, the complications of diabetes are also worth exploring. Regarding the most significant microvascular complications of diabetes, this study found a causal relationship between hypothyroidism and type 1 diabetes with renal complications but not with type 2 diabetes with renal complications. Our results are in agreement with many previously reported studies (30). Diabetic kidney disease is the leading cause of end-stage renal failure. Given the diversity of cell populations within the kidney and the various physiological roles of the kidney, the development of diabetic kidney disease is complex (31), and it is difficult to determine the mechanisms by which hypothyroidism plays a role. However, there is growing evidence that hypothyroidism has detrimental effects on renal structure during development (32) and adulthood (33, 34), including altered renal weight ratio (35) and reduced area of glomerular basement membrane thickening and expansion of the thylakoid matrix (36, 37). Hypothyroidism directly or indirectly exacerbates renal dysfunction in the diabetic state, including 1. altering the renin-angiotensin-aldosterone system (RAAS) (38–41) and affecting renal tubular ion transport (42–44). 2. affecting renal basement membrane outer chloride channel protein expression, resulting in impaired tubulo-glomerular feedback (39). 3. affecting cardiac output. 4. decreasing synthesis and activity of renal vascular dilators (i.e., nitric oxide, adrenomedullin) leads to intrarenal vasoconstriction, which reduces renal perfusion (45). A large body of evidence also suggests that inflammation plays a key role in the development and progression of diabetic complications (46). Immune response cells including innate and adaptive immunity are involved in diabetic nephropathy (47). Inflammatory factor expression and immune response triggered by hypothyroidism also work in the development of diabetic nephropathy. In summary, hypothyroidism leads to a decrease in glomerular filtration rate (GFR), renal plasma flow (RPF) and glomerular transcapillary hydrostatic pressure, aggravating renal dysfunction in the diabetic state (48, 49). Furthermore, there is a causal relationship between hypothyroidism and both nonproliferative and proliferative retinopathy. Thyroxine is an important regulator of neurodevelopment. In the retina, TH is essential for retinal development, photoreceptor differentiation and cone optin expression and regulates photoreceptor survival in healthy and disease conditions (50). In diabetes, the internal blood retinal barrier is the first to be damaged (51), combined with a decrease in thyroid hormones, further aggravating retinal damage. In addition, it has been found that elevated serum thyrotropin levels are associated with retinal small artery stenosis (52). Our study further confirms that hypothyroidism has an important driving role in diabetic retinopathy.

In this study, the causal relationship between hypothyroidism and diabetes mellitus and its microvascular complications was investigated by Mendelian randomization. The appropriate GWAS database was selected from a genetic perspective. Multiple analyses were also used, and the robustness and sensitivity of the data were considered. Of course, there are some limitations of this study. Regarding the staging of diabetic retinopathy, this study only started from proliferative and nonproliferative and did not subdivide whether it was type I or type II. Future studies can go deeper in this section.

In conclusion, the study reveals the causal relationship between hypothyroidism and diabetes and its microvascular complications. With the increase in hypothyroidism, the risk of developing type 1 diabetes, type 1 diabetes with renal complications, severe nonproliferative diabetic retinopathy and proliferative diabetic retinopathy increases. However, there was no effect on type 2 diabetes and type 2 diabetes with renal complications. This study provides new insights into the prevention and treatment of diabetes and its microvascular complications, suggesting that hypothyroidism is a risk factor for new-onset diabetes and antithyroid drugs can worsen diabetes mellitus and its microvascular complications. This will guide the clinical use of medications in patients with hyperthyroidism and suggest the importance of diabetes screening in patients with hypothyroidism.

## Data availability statement

The datasets presented in this study can be found in online repositories. The names of the repository/repositories and accession number(s) can be found in the article/**Supplementary Material**.

## Author contributions

TF: Conceptualization, Methodology, Software, Writing – original draft. XD: Data curation, Investigation, Writing – original draft. JW: Methodology, Visualization, Writing – original draft. FH: Methodology, Resources, Supervision, Writing – original draft. XL: Investigation, Resources, Writing – original draft. YL: Data curation, Methodology, Software, Writing – original draft. BS: Writing – review & editing. LC: Writing – review & editing.
