# Supplementary material for: The effect of hypothyroidism on the risk of diabetes and its microvascular complications: a Mendelian randomization study
Source: Front Endocrinol (Lausanne). 2023 Dec 5;14:1288284. doi: 10.3389/fendo.2023.1288284 (PMC10728873; doi:10.3389/fendo.2023.1288284)
Supplement: Supplementary file 1 [file DataSheet_1.docx]

**Supplementary Material**

**
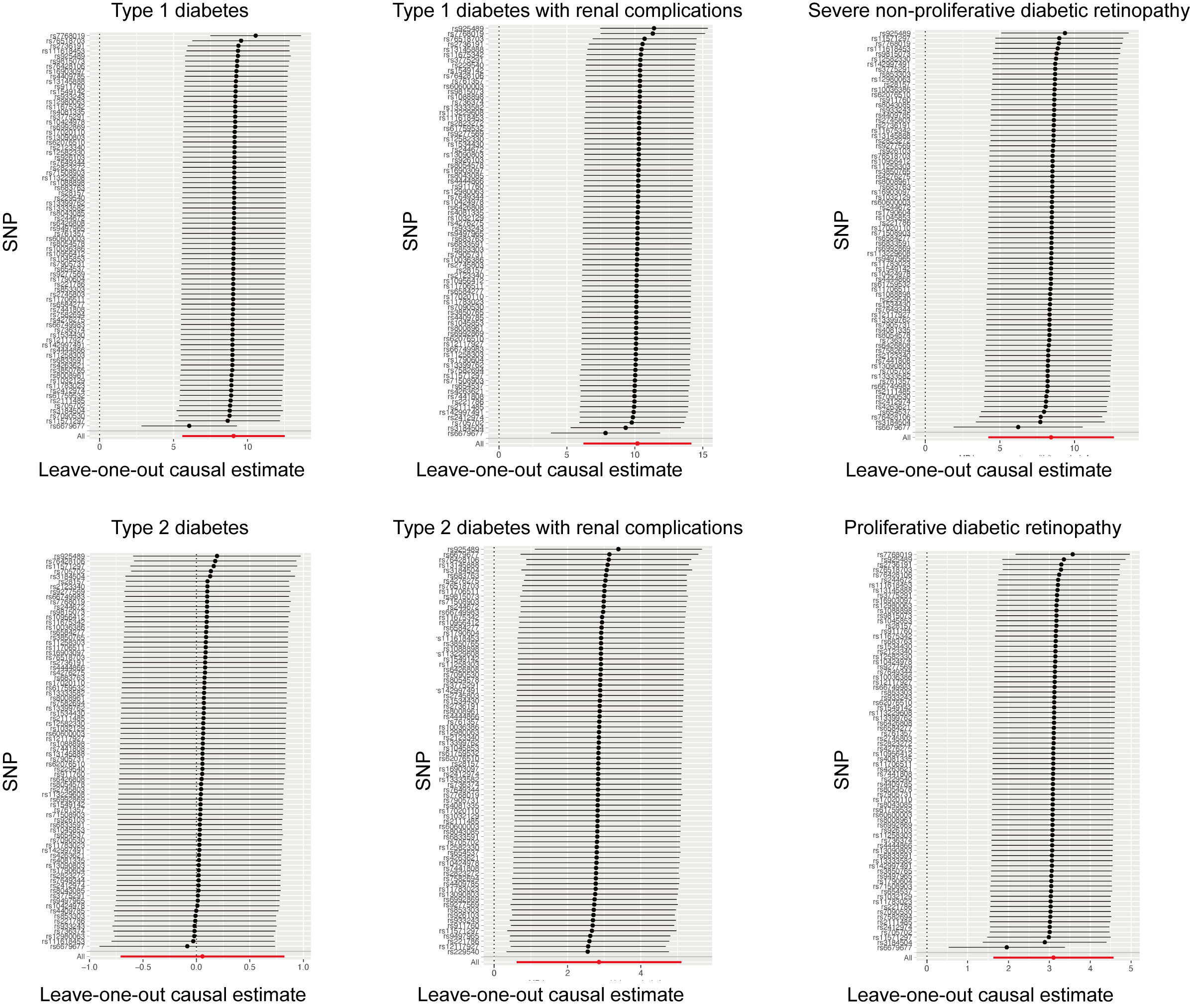
**

**Figure S1**

Leave-one-out plots of the causal association between hypothyroidism and diabetes mellitus and its microvascular complications.

**
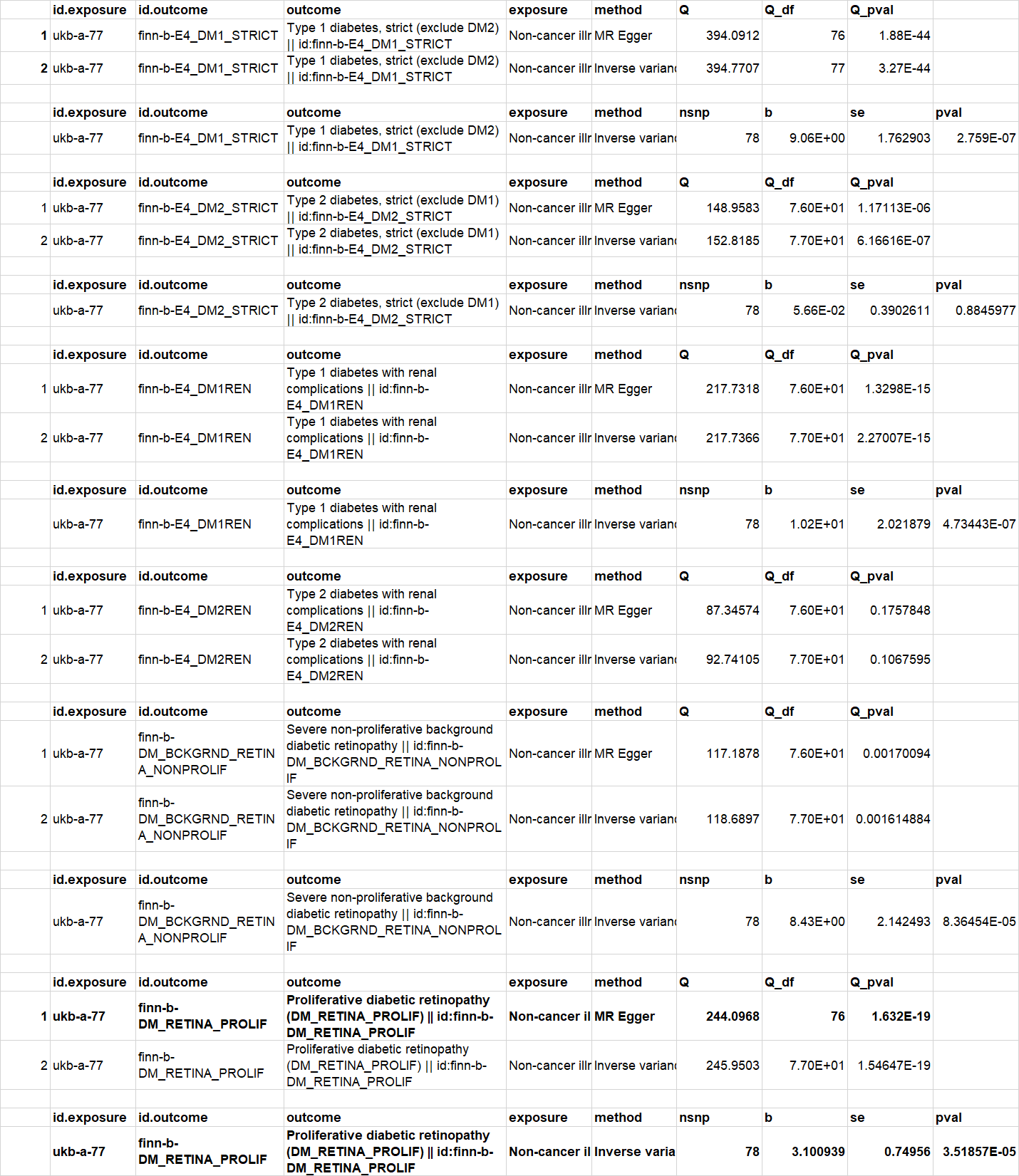
**

**Table S1**

Heterogeneity test estimates for the MR result. Inverse variance weighted (multiplicative random effects) was used to estimate the MR effect size when Q pval much less than 0.05.


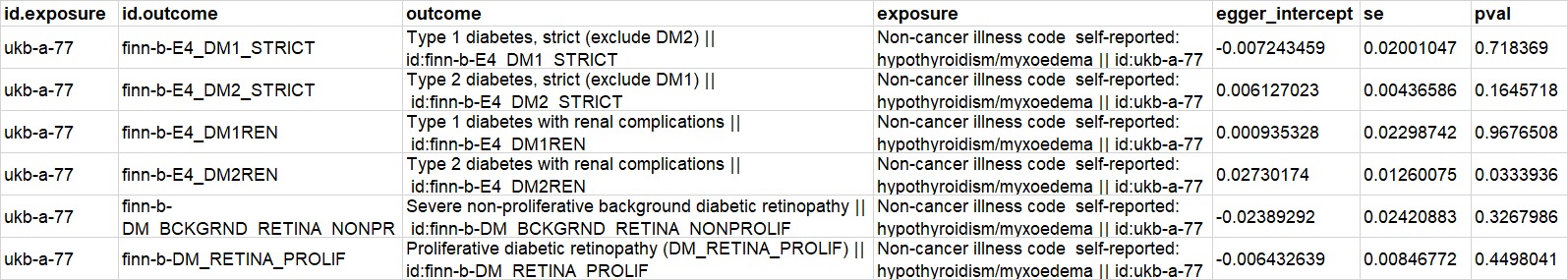


**Table S2**

Pleiotropy test estimates for the MR result.
